# DeepSpot: A deep neural network for RNA spot enhancement in single-molecule fluorescence in-situ hybridization microscopy images

**DOI:** 10.1017/S2633903X22000034

**Published:** 2022-04-19

**Authors:** Emmanuel Bouilhol, Anca F. Savulescu, Edgar Lefevre, Benjamin Dartigues, Robyn Brackin, Macha Nikolski

**Affiliations:** 1 CNRS, IBGC, UMR 5095, Université de Bordeaux, Bordeaux, France; 2 Bordeaux Bioinformatics Center, Université de Bordeaux, Bordeaux, France; 3 IDM, Faculty of Health Sciences, University of Cape Town, Cape Town, South Africa; 4 Advanced Medical Bioimaging CF, Charité—Universitätsmedizin, Berlin, Germany

**Keywords:** Deep learning, mRNA spot detection, smFISH image analysis

## Abstract

Detection of RNA spots in single-molecule fluorescence in-situ hybridization microscopy images remains a difficult task, especially when applied to large volumes of data. The variable intensity of RNA spots combined with the high noise level of the images often requires manual adjustment of the spot detection thresholds for each image. In this work, we introduce DeepSpot, a Deep Learning-based tool specifically designed for RNA spot enhancement that enables spot detection without the need to resort to image per image parameter tuning. We show how our method can enable downstream accurate spot detection. DeepSpot’s architecture is inspired by small object detection approaches. It incorporates dilated convolutions into a module specifically designed for context aggregation for small object and uses Residual Convolutions to propagate this information along the network. This enables DeepSpot to enhance all RNA spots to the same intensity, and thus circumvents the need for parameter tuning. We evaluated how easily spots can be detected in images enhanced with our method by testing DeepSpot on 20 simulated and 3 experimental datasets, and showed that accuracy of more than 97% is achieved. Moreover, comparison with alternative deep learning approaches for mRNA spot detection (deepBlink) indicated that DeepSpot provides more precise mRNA detection. In addition, we generated single-molecule fluorescence in-situ hybridization images of mouse fibroblasts in a wound healing assay to evaluate whether DeepSpot enhancement can enable seamless mRNA spot detection and thus streamline studies of localized mRNA expression in cells.

## Impact Statement

Our paper introduces DeepSpot, a Deep Learning-based tool specifically designed to enhance RNA spots which enables downstream spot detection without the need to resort to image per image parameter tuning. DeepSpot’s architecture is inspired by small object detection approaches by integrating dilated convolutions into a module specifically designed for context aggregation for small object and using Residual Convolutions to propagate this information along the network.

## 1. Introduction

Single cell microscopy together with RNA single-molecule fluorescence in-situ hybridization (smFISH) technologies allows gene expression profiling at subcellular precision for determining molecular states of various cell types^(^^1^ ^)^ and that in high-throughput fashion^(^^2^ ^)^. The repertoire of mRNA expression quantification methods is large and includes smFISH, clamp-FISH, amp-FISH, and multiplexed versions, such as MerFISH, all allowing the localization of RNA at subcellular level. There are technological differences between these methods in terms of number of detected RNAs and number of processed cells; however, all produce imaging data with mRNA spots that can be further matched to the spots’ *x* and *y* coordinates. With such increased image acquisition automation and the consequent growing number of high-throughput projects focused on spatially resolved transcriptomics, the need for automated and highly accurate detection of mRNA spots in fluorescent microscopy images has become increasingly important.

smFISH is a method for visualizing individual RNA transcripts in fixed cells. smFISH is based on targeting RNA molecules with a set of 24–48 oligonucleotide probes, each individually labeled with one fluorophore. The combined fluorescence intensity level obtained from this high number of probes makes each RNA transcript visible as a spot that can be computationally identified and quantified^(^^3^ ^)^.

Despite the progress made in recent years, it is still difficult to detect the localization of spots corresponding to different mRNAs in a fully automated manner. First, background intensity is often irregular due to various factors, including autofluorescence that can be caused by intrinsic cell properties and fixative-induced fluorescence phenomenon^(^^4^ ^)^ or growth medium and buffers. An additional contributor to the background noise is off-target binding of probes, which depends on a various number of parameters, including the length of the transcript, its sequence, the cell type used, and others. Second, spot detection is affected by the nonhomogeneous intensity distribution and indistinct spot boundaries relative to the background. Moreover, fluorescence in-situ hybridization (FISH) images may have a low signal-to-noise ratio (SNR). Additionally, the boundary between background (noise) and signal (spots) is usually not evident^(^^5^ ^)^.

The main drawback of classical mRNA spot detection methods is the requirement of a strong human input to determine the best parameters to handle variable image to image properties such as SNR and presence of artifacts. Even small differences in these characteristics lead to the necessity for parameter fine-tuning^(^^6^ ^)^. Other than being time-consuming, the quality of detection largely depends on the capacity of the user to correctly choose the method’s parameters according to each image properties (contrast, spots, artifacts, and noise). Some recent deep-learning-based approaches for mRNA spot detection try to circumvent this limitation, such as deepBlink^(^^7^ ^)^.

Here, we introduce DeepSpot, a convolutional neural network (CNN) method dedicated to the enhancement of fluorescent spots in microscopy images, thus enabling downstream mRNA spot detection by conventional widely used tools without need for parameter fine-tuning. With DeepSpot, we show that it is possible to avoid the manual parameter tuning steps by enhancing the signal of all spots so that they have the same intensity throughout all images regardless of the contrast, noise, or spots shape. All the code as well as the pretrained model is available on GitHub, and a plug-in for the image analysis tool Napari is also distributed (https://github.com/cbib/DeepSpot).

DeepSpot gives a new twist to the residual network (ResNet) network architecture and learns to automatically enhance the mRNA spots, bringing them all to the same intensity. In parallel, a multinetwork architecture is integrated, trained by minimizing the binary cross-entropy (BCE) while providing context for mRNA spots thanks to the atrous convolutions. We evaluated the impact of the spot enhancement on the downstream mRNA spot detection, by performing spot detection using Icy with fixed parameters on both simulated images and experimental images manually annotated. Moreover, we compared the quality of mRNA spot detection from images enhanced by DeepSpot with deepBlink, and have shown that our method achieves greater generalization to effectively handle full variability of smFISH data. Finally, to illustrate the end-to-end use of DeepSpot in projects where detecting subcellular localization with high precision is essential, we generated smFISH images from mouse fibroblasts in a wound healing assay, where enrichment of expression of 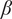 *-Actin* toward the location of the wound is expected in the migrating 3T3 mouse fibroblasts.

## 2. Related Work

Methodologically mRNA spot detection can be related to the detection of small objects topic in image analysis. The goal of spot detection is to find small regions in an image that differ from the surroundings with respect to certain properties, such as brightness or shape, more precisely, regions with at least one local extremum^(^^8^ ^)^. Spots can be considered as a particular case of more or less circular objects of small extent. Object detection has been one of the key topics in computer vision which goal is to find the position of objects. However, small object detection, such as mRNA spots, remains difficult because of low-resolution and limited pixels^(^^9^ ^)^.

In this work, we propose a deep learning network inspired by small object detection approaches for mRNA spot enhancement and we show how it can enable the downstream accurate detection of spots.

### 2.1. mRNA spot detection

**Figure 1.**
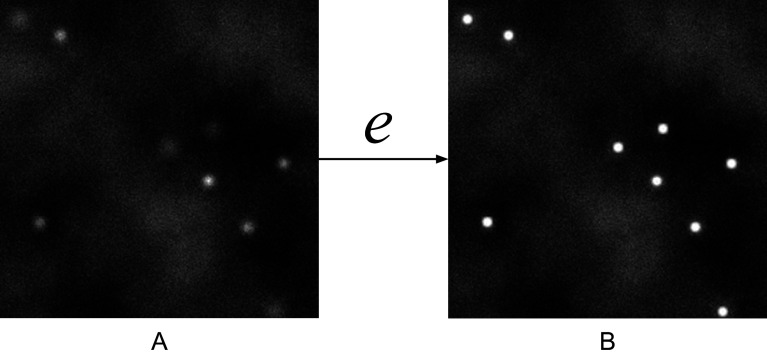
(a) RNA spots on a noisy background. (b) Spots’ intensity is increased after the enhancement by 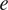 .

In the case of FISH images, mRNA spots are small, compact, and smaller than the resolution limit of the microscope^(^^10^ ^)^; therefore, images of mRNA spots correspond to the maximum intensity pixel surrounded by the diffraction of the fluorescence signal defined by the point spread function (PSF), which can be modeled by a Gaussian within small radius disk (see Figure 1b). This radius depends on several imaging parameters and optical properties of the microscope such as the diffraction limit or the excitation state of the fluorophore.

While there is no universal solution to the detection of small objects such as mRNA spots in fluorescent cellular images, a large plethora of work is available on the subject. A number of approaches have gained wide popularity thanks to the development of software tools embedding the algorithms and providing user with a graphical interface. In particular, ImageJ/Fiji^(^^11^ ^,^^12^ ^)^ is widely used, largely due to the plug-in-based architecture, recordable macro language, and programmable Java API. Another popular tool is CellProfiler^(^^13^ ^)^, based on similar paradigms and FISH-Quant^(^^14^ ^)^. A more recent platform, Icy^(^^15^ ^)^, also provides the possibility to develop new algorithms as well as a user interface for image analysis, including the Icy spot detector for the detection of mRNA spots, a method based on wavelet transform decomposition^(^^10^ ^)^.

Deep learning networks have been introduced for mRNA spot detection^(^^16^ ^,^^17^ ^)^, and more recently deepBlink^(^^7^ ^)^. The latter focuses on a fully convolutional neural network based on the U-Net architecture. deepBlink not only provides the code, but also annotated smFISH data and implements a threshold-independent localization of spots.

### 2.2. Detection and enhancement of small objects

Consistently with the mRNA spot detection difficulty, detection of small objects remains a challenging part of the general object detection problem due to the limited information contained in small regions of interest. For instance, it has been shown that the object size has a major impact on the accuracy of Deep Learning object detection networks such as VGG, ResNet, or Inception V2^(^^9^ ^)^. Indeed, small objects do not contain sufficient semantic information^(^^18^ ^)^, and thus the challenge is to capture semantic features while minimizing spatial information attenuation^(^^19^ ^)^.

Expectedly, adding more context improves the detection of small objects^(^^19^ ^–^^21^ ^)^. An elegant solution is to use the dilated convolution (a.k.a. atrous convolution), because the receptive field can be expanded without loss of resolution and thus capture additional context without loss of spatial information^(^^22^ ^,^^23^ ^)^.

Of particular interest to our work is the signal enhancement, an image processing technique aiming to reinforce the signal only in those regions of the image where the actual objects of interest are, and potentially to weaken the noise or the signal from other structures^(^^24^ ^)^. In our case, the objects of interest are mRNA spots.

Image enhancement is the transformation of one image 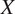 into another image 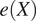 (see Figure 1). Pixel values (intensities) of 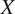 at spot locations are modified according to the transformation function 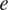 , the resulting pixel values in image 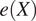 measuring the certainty of mRNA presence at that position. Thus, 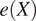 can be considered as a probability map that describes possible mRNA spot locations^(^^24^ ^)^.

Small object enhancement has been developed in other fields than mRNA spots in fluorescent imaging, such as in astronomy to enhance stars or galaxies over the cosmic microwave background^(^^25^ ^)^ or in the biomedical imaging to facilitate the human detection of larger spots such as nodules^(^^26^ ^)^. In the microscopy field, Deep-STORM^(^^27^ ^)^ enhances the resolution of single-molecule localization microscopy mRNA spots using deep learning. However, these images do not have the same characteristics as smFISH data in terms of noise or signal. For example, human nodules are much larger objects than typical mRNA spots. As for the star enhancement method proposed by Sadr *et al.* ^(^^25^ ^)^, it is not suited for low-intensity spots^(^^28^ ^)^, which is a major concern in spot detection for smFISH images.

## 3. Materials and Methods

### 3.1. Materials

#### 3.1.1. smFISH datasets with alternatively established spots’ localization

We constructed a dataset 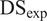 of 1,553 images from the experimental smFISH data acquired in Reference (29), by applying 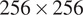 pixels patches to better fit in the GPU memory (see Table 1). The authors have performed spot detection using image analysis techniques, such as local maximum detection, implemented in the BIG-FISH pipeline^(^^30^ ^)^. Along with the scripts, the authors provided a list of 57 parameter combinations that were used to detect the spots in 57 different image acquisition series of 32 genes used in their study. We ran the pipeline with these parameters and performed an additional manual curation to keep patches with number of spots between 10 and 150 and remove those with a visually obvious over- or under-identification of spots. The resulting 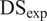 dataset of 1,553 images representing 27 different genes is thus experimentally generated and guarantees high confidence in the ground truth annotation of mRNA spot coordinates.

**Table 1.** List of datasets used for training (22 training datasets) and evaluation (21 test datasets) of the DeepSpot network. Images in the 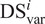 datasets have spot intensities between 160 and 220 for each image. For the fixed intensity datasets 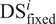 , the spot intensity is set to one value within [160…220] for a given image, but varies from image to image. Ten variable intensity 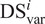 and 10 fixed intensity 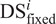 datasets are named according to the number of spots in the images, 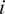 . The dataset 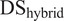 combines 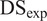 with 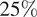 simulated images.

Datasets	#Spots per image	Intensity	#Images train	#Images test	#Datasets train/test
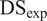	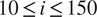	Variable	1,453	100	1/1
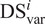	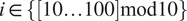	Variable	2,000	400	10/10
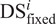	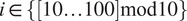	Fixed	2,000	400	10/10
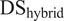	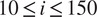	Variable	2,000	NA	1/NA

We have also downloaded the dataset 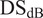 from the deepBlink publication^(^^7^ ^)^, composed of 129 smFISH images acquired from four different cell culture conditions of the HeLa-11ht cell line. The authors provided their annotation of mRNA spots’ locations, which was performed using TrackMate and curated by experts.

To estimate the variability of spot intensity, we also calculated the coefficient of variation (CV) of spot intensities for each gene of 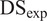 as well as for the entire 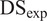 and 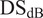 datasets (Supplementary Figure S1). While, for most images and genes, the CV ranges from 0.25 to 0.50, some have a very high CV. The global CV for all the spots of all the images in the 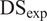 dataset is 0.72 and 0.22 for 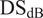 .

#### 3.1.2. Novel smFISH dataset from a wound healing assay

To evaluate whether DeepSpot enables precise mRNA spot detection in a biological context, we made use of the wound healing assay^(^^31^ ^)^ to generate a second experimental dataset 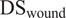 . In the wound healing assay, migrating cells, such as fibroblasts, are grown on a coverslip and serum starved for synchronization. A scratch in the middle of the coverslip is then generated, mimicking a wound, followed by induction of the cells to polarize and migrate toward the wound, to generate wound closure, done using replacement of serum starved medium with 10% FBS-containing medium. We used 3T3 mouse fibroblasts in a wound healing assay, followed by cell fixation. Fixed samples were taken for smFISH experiments to visualize and quantify 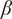 *-Actin* mRNA and imaged on a custom-built Nikon Ti Eclipse widefield TIRF microscope. 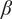 *-Actin* has been previously shown to be enriched in neuronal growth cones of extending axons, as well as the leading edges of migrating cells, and this enrichment has typically been associated with cell polarity and neuronal plasticity^(^^32^ ^–^^34^ ^)^. Based on this, we hypothesized that 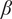 *-Actin* would be enriched in the leading edge of migrating 3T3 fibroblasts. The dataset is composed of 96 images, 48 images of nonmigrating cells (control), and 48 images of migrating cells. Each image was divided into four patches, yielding a total of 384 patches of 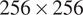 pixels size.

#### 3.1.3. Simulated and hybrid datasets

In addition to the experimental datasets, we have built 20 simulated datasets with images 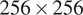 pixels of width and height, the same as the patches of 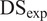 . Briefly, the background was generated by a combination of Poisson noise and Perlin noise with a random intensity between [80, 150]. Elastic transformations were added to this noise to approximate the variety of textured background noise in the experimental images. Spots were generated as circles, randomly placed in the image, and then convolved with a Gaussian function that approximates PSF. Their size randomly ranges from 4 to 9 pixels in diameter, including Gaussian smoothing.

Two different types of simulated datasets were generated 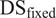 and 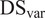 , each containing 10 datasets defined according to the number of spots per image 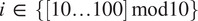 (see Table 1). For example, in the 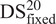 dataset, each image contains 20 spots, and in the 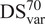 dataset, each image contains 70 spots. Each image in the 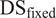 dataset has the same fixed spot intensity for all spots randomly chosen in the interval [160, 220], whereas in the 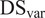 dataset, the spot intensity is randomly chosen from the same interval for each spot, resulting in images with variable spot intensity.

In addition to the experimental and simulated datasets, we have built a hybrid dataset 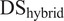 where the experimental data from 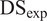 are augmented by appending an additional 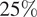 of simulated images generated with both variable spots’ intensity and variable spots’ number per image, within the [160, 220] and [10, 100] intervals, respectively. The intensity of the spots is calculated with respect to the intensity of the background noise, so that the generated images have an SNR between 10 and 40. These values correspond to the minimum and median SNR values in our experimental images, respectively (Supplementary Figure S2).

#### 3.1.4. Ground truth

Since the goal of our network is to learn to transform an image 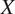 into 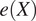 , where intensity at spots’ location is enhanced, the training step has to be provided with the enhanced counterpart of each image in the training set. That is, training sets include pairs of images 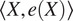 where 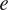 is the procedure that is used to produce ground truth enhanced images: for each spot (Figure 1a), the ground truth enhancement procedure 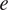 is applied at the spots’ locations 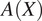 , resulting in images where spots are enhanced as shown in Figure 1b.

In this work, we implement 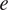 as a kernel of 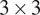 pixels at all locations where the spots were annotated in the experimental dataset 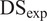 or generated for 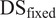 , 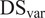 , and 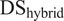 (see Table 1). The kernel has the same pixel values for all the spots, in order to drive the network to learn to enhance all spots up to the same level of intensity, regardless of the initial intensity in the acquired data. The enhancement kernel of DeepSpot is smaller than the smallest spot size in our datasets; therefore, it is not expected to augment the size spot. This is particularly important for spatially close spots. Moreover, background is kept the same between 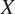 and 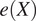 , so the transformation does not affect the background.

### 3.2. Method

In this section, we present the DeepSpot enhancement network in detail. We first overview the network architecture and then we discuss the custom loss function.

#### 3.2.1. Network architecture

**Figure 2.**
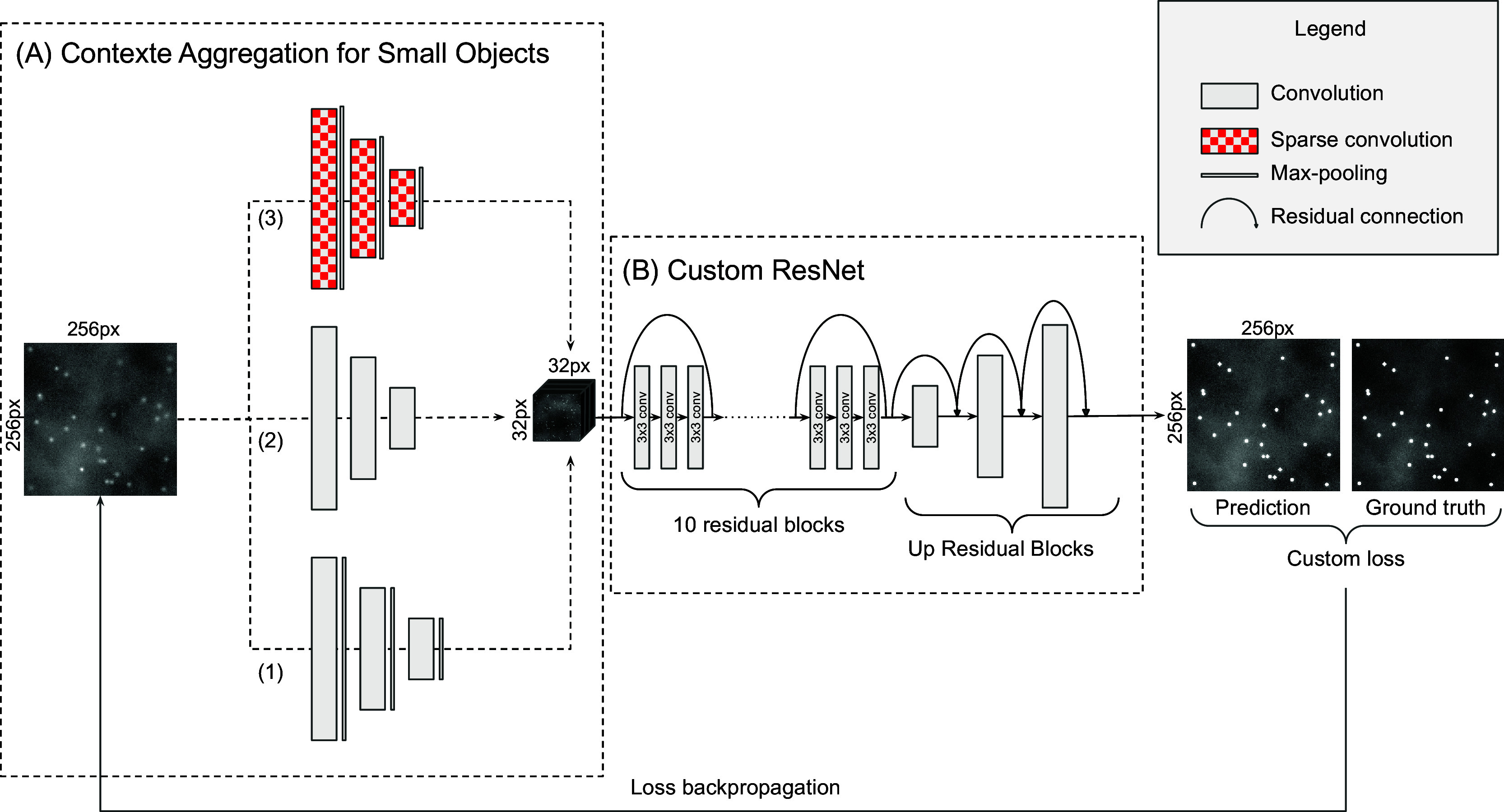
DeepSpot network architecture is composed of the context aggregation for small objects module constituted of a multipath network (Panel A) and a customized ResNet component (Panel B). A custom loss function is used for training the network.

DeepSpot network is composed of two main components as shown in Figure 2. The first component is a multipath network shown in Panel A. The second component is an adapted residual network as shown in Panel B.

##### Context aggregation module

As pointed out in Reference (22), finding small objects is fundamentally more challenging than large objects, because the signal is necessarily weaker. To solve this problem in the context of mRNA spot detection, we developed a new module, that we called the context aggregation for small objects (CASO) module^(^^35^ ^)^, demonstrated that using image evidence beyond the object extent (context) always enhances small object detection results, and we therefore developed our CASO module to aggregate context around the mRNA spots. The CASO module is a multipath network as shown in Panel A of Figure 2. It takes the input image and processes it along three different paths each with different types of convolution blocks to collect specific information from the input image. Each path contains three convolution blocks.

1. The first path is composed of traditional convolution blocks (2D convolution, batch normalization, activation, and max pooling). These blocks, often used in CNNs, are particularly efficient to reinforce the semantic information at the expense of spatial information.
2. The second path uses only 2D convolutions, batch normalization, and activation. As Max Pooling is known to keep mostly the maximum intensities in images, some of the faint spots may be eliminated during the max pooling operation. In this path, we used strided 2D convolutions instead of a max pooling layer, to keep the information of low intensity of spots. To make sure to end up with the same receptive field as the first path, we set the stride to 2.
3. The third path makes use of the atrous convolution pooling^(^^22^ ^)^, implemented as a 2D convolution with the dilatation rate of 2. The following layers are batch normalization, activation, and max pooling.

The CASO module is a multipath neural network and can learn more comprehensive and complementary features than a single path. In particular, the goal of the atrous convolution is to bring more context around the small spots (see Section 2.2), while the two other paths aggregate the semantic information of the bright spots and faint spots for the first and second paths, respectively. The results of the three encoding paths are then concatenated to construct a longer feature vector containing information extracted by each path. For all convolutional blocks, the activation function is the rectified linear unit (ReLU). The number of filters for the 2D convolutions in the CASO module are 32, 64, and 128 for the first, second, and third paths, respectively.

##### Custom ResNet

**Figure 3.**
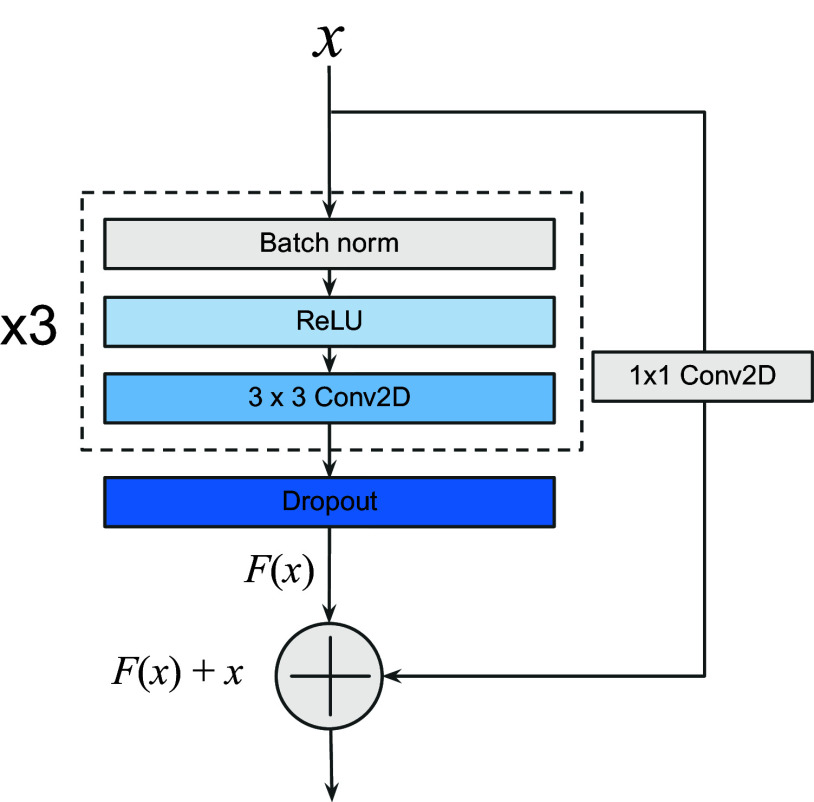
Full pre-activation residual block, composed of batch normalization, activation, and convolution, repeated three times before dropout and residual connection.

For the second component (Panel B of Figure 2), we customized the ResNet architecture to create a residual neural network composed of 10 consecutive convolutional residual blocks (ResBlock), using full pre-activation blocks described in Reference (36), where the authors suggested that the better results obtained by the full pre-activation blocks are due to the pre-activation by the batch normalization that improves regularization of the model due to the fact that the inputs to all weight layers have been normalized. Each ResBlock is composed of three subblocks as shown in Figure 3. A subblock is constituted of a batch normalization followed by an activation (ReLU) and a 2D convolution. After the three subblocks, a spatial dropout layer with the rate of 0.2 is applied. Each ResBlock ends by the residual connection.

To obtain an output image with the same size as the input image, we used a particular type of convolutional block. Recently, Reference (37) demonstrated that the use of up residual blocks (UpResBlocks) instead of classic up-convolution blocks improves the performance of generative networks by preserving the effective features from the low-dimensional feature space to the high-dimensional feature space. Our decoding path is composed of three UpResBlocks and reconstitutes an output with the same size as the input while propagating low-dimensional feature information of the enhanced spots from the custom ResNet to the last layer. Each UpResBlock is constituted of three subblocks containing a 2D transposed convolution, batch normalization, and activation (ReLU). The three subblocks are followed by a spatial dropout layer with the rate of 0.2. UpResBlocks then end with a residual connection. A sigmoid activation function is applied to the last convolution, so that all the pixels have values in the 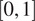 interval. The final image is obtained by normalizing the pixel intensities between 0 and 255.

#### 3.2.2. Loss

We defined our custom loss function as a combination of BCE and mean squared error (MSE) functions. The main term of the loss function is the BCE loss, defined by 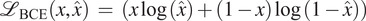 that measures the difference between the images predicted by the network 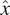 and the ground truth images 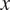 . While mostly used for classification, it can also be used for segmentation and enhancement due to its performance for pixel-level classification^(^^38^ ^)^.

To this main 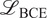 term, we added a regularization term defined by MSE, 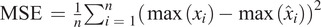 that is computed between the maximum value of the predicted image 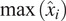 and the maximum value of the ground truth image 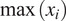 . This regularization drives the network to produce spots whose intensity is close to 255 (see Table 2), and therefore standardizes the signal enhancement intensity in the output images, which in its turn facilitates the downstream automatic detection of the spots. The total loss function is 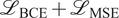 .

**Table 2.** Spot enhancement performance in terms of resulting spot intensity. The measures displayed correspond to the spot intensity between [0, 255] after enhancement by the neural network and averaged by category of models and datasets. Between brackets are shown the 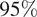 confidence intervals. Model categories are listed in rows, whereas columns correspond to the dataset categories on which the different models were applied.

Test Train	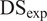	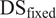	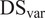
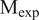	249.55	250.81	247.07
	[249.29, 249.82]	[250.68, 250.93]	[246.68, 247.45]
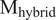	241.15	248.71	243.3
	[240.79, 241.52]	[248.57, 248.85]	[243.14, 243.45]
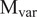	248.92	251.99	254.57
	[248.47, 249.37]	[251.93, 252.05]	[254.53, 254.62]
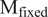	242.93	254.73	249.33
	[242.16, 243.71]	[254.71, 254.75]	[249.09, 249.57]

## 4. Results

We trained the DeepSpot network on our 20 simulated training datasets 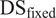 and 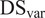 as well as on the experimental and hybrid datasets 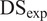 and 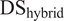 , resulting in 22 models 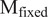 , 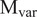 , 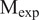 , and 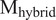 . Training parameters were optimized with HyperOpt algorithm^(^^39^ ^)^ and the ASHA scheduler^(^^40^ ^)^. The best configuration obtained and used for further trainings had the learning rate of 0.0001, the dropout rate of 0.2, and the batch size of 32 and 128 filters per convolution.

Each of the resulting 22 models was evaluated on the 21 test datasets from Table 1, yielding 462 enhanced test datasets (8,100 images in total). To assess whether DeepSpot enhancement enables easy spot detection, we applied the Icy spot detector ^(^^10^ ^)^ to the images enhanced by different models. Moreover, we defined a unique set of Icy parameters that matches the shape and intensity of the enhancing kernel of the DeepSpot network: scale 3 and sensitivity 20, and scale 7 and sensitivity 100. We then evaluated whether the detected spots from the enhanced images matched well with the annotated ground truth of spots’ coordinates.

### 4.1. Evaluation procedure

We denote by 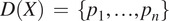 the point pattern detected by Icy from the enhancement of an image 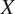 by DeepSpot and by 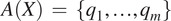 the ground truth annotation of spot coordinates. Notice that 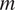 is not necessarily equal to 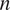 , corresponding to under- or over-detection, and even for a well-detected spot, the coordinates in 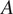 and 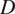 may slightly differ. To account for these remarks, we used the 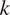 -d tree algorithm^(^^41^ ^)^ to query the detection 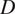 for nearest neighbors in 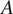 as proposed in References (42,43). The number of neighbors was set to 1 and the matching radius 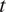 to 3 (coherent with the enhancement kernel for ground truth images).

This allows to establish a matching for all annotated points under 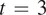 and thus also defines the number of False Negatives or False Positives corresponding to the missing matches from 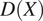 or 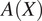 , respectively. True Negatives are defined by all pixels 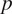 of the confusion matrix such that 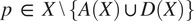 . However, given that 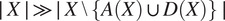 implies inflated TN values, this makes measures such as accuracy, AUC, and ROC curve irrelevant.

**Figure 4.**
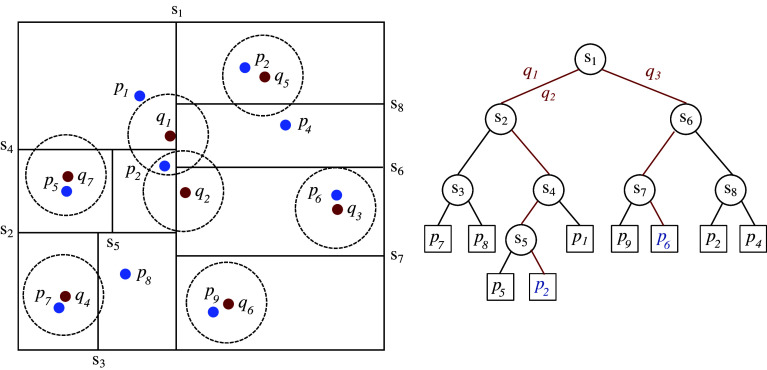
Spot matching by 1-neighbor 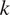 -d tree between the detected mRNA spots 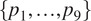 depicted in blue and annotated spots 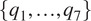 depicted in red. The 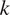 -d tree construction for 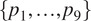 is shown on the left. Using the matching radius depicted by circles, the 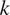 -d tree queries for 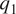 and 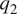 , shown in red, lead to the same leaf 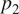 and correspond to an ambiguous match, while query for 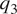 leads to an unique match. mRNA spots 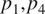 , and 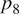 are the False Negatives.

The drawback of matching 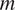 versus 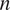 points is the possibility of ambiguous matching. With the 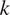 -d tree approach, it happens when two points 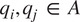 can match to one 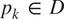 (see Figure 4). This can happen if annotated spots 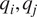 are close and the detected matching point for both of them, 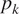 , lies within the same distance 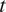 , which can correspond to an over-enhancement and thus blurring between the two spots in the enhanced image. While alternative solutions such as the Linear Assignment Problem can be used, they do not avoid the problem of matching two different numbers of points. The 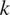 -d tree approach has the advantage to keep the ambiguous matches (AMs) explicitly to measure this effect. We thus also report the number of AMs.

### 4.2. DeepSpot enhances the mRNA spot signal to the same intensity

To avoid the manual selection of the detection threshold, it is imperative to have a homogeneous spot intensity for the whole dataset in order to use a unique set of parameters for all images. Table 2 summarizes the intensities obtained after enhancement by DeepSpot for each category of datasets described in Table 1 (experimental, hybrid, simulated with variable, and fixed intensities).

As expected, intensities were closer to 255 when training and test datasets belong to the same category. For example, models trained on data with fixed intensities 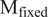 and applied to data with fixed intensities 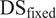 produced enhanced spot intensities very close to 255. Similarly, enhancement close to 255 could be observed when models 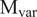 were evaluated on 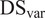 . Of particular interest are the enhancement results from the 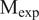 training that are close to 250 for every dataset, experimental or simulated. Hybrid model enhances intensities to 241 on the experimental dataset. In general, the enhanced spot intensities were between 241 and 255, representing a variation of only 5.4% from the maximum intensity, which is sufficient to fully separate smFISH spots from the background in the enhanced images.

### 4.3. DeepSpot enables accurate mRNA spot detection

The summary statistics of performance of each model type are reported in Table 3 including the mean F1-score, precision, recall, and AMs, with 95% confidence interval. For a given model 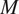 , each metric was computed for all enhanced images (8,100 in total). The mean metric value 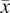 and 95% confidence interval 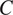 were then calculated separately for each model type. Due to the high prevalence of True Negatives, instead of the Accuracy measure, we calculated the F1-score, which gives an indication of the model accuracy with a better balance between classes than the actual accuracy measure, by not including True Negatives. We compared the F1-scores for each of the 14 genes present in the test dataset; no major difference in DeepSpot performance can be observed for these genes (Supplementary Figure S3). This consistency in performance across different SNR values (Supplementary Figure S2) shows that DeepSpot has the capacity to enhance spots in images with varying characteristics.

**Table 3.** Models’ performance per model type. Metrics (F1-score, precision, recall, and ambiguous matches [AMs]) were calculated by averaging the values obtained for each image of the 21 test datasets. Top values in cells correspond to the mean value, whereas bottom values between brackets show the 95% confidence interval. Best values are highlighted in bold.

Model	F1-score (%)	Precision (%)	Recall (%)	AM (mean/img)
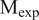	87.8	99.6	80.1	2.7
	[87.6, 88.1]	[99.5, 99.7]	[79.8, 80.5]	[1.4, 4.0]
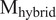	**97.3**	99.3	**95.5**	**0.94**
	[97.2, 97.3]	[99.2, 99.4]	[95.4, 95.6]	[0.56, 1.32]
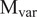	94.3	99.5	91.4	1.19
	[94.3, 94.4]	[99.5, 99.5]	[91.3, 91.5]	[1.03, 1.35]
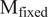	91.6	**99.9**	85.7	2.05
	[91.5, 91.7]	[99.9, 99.9]	[85.6, 85.8]	[1.77, 2.32]

The results in Table 3 indicate that the number of FP is very low for all models, given that both precision and recall are high. Importantly, 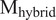 has shown best overall performance in terms of precision, recall, and F1-score, thus indicating that the mRNA spot enhancement by 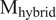 leads to the least FP and FN counts in the downstream mRNA spot detection.

**Figure 5.**
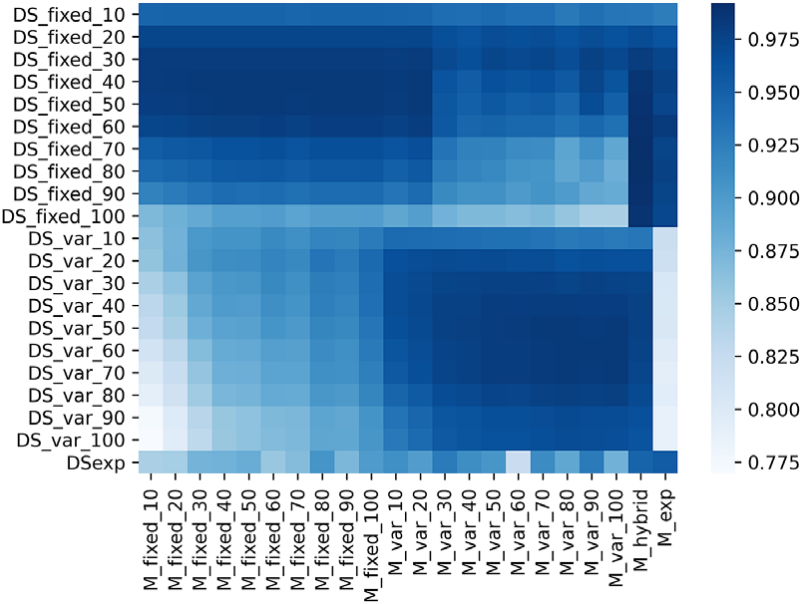
Heat map of the F1-scores obtained by each of the 22 models when evaluated on the 21 test datasets described in Table 1.

Figure 5 shows the mean F1-scores for each of the 22 models evaluated on the 21 datasets. This heat map indicates that (a) models trained on the 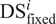 training sets perform better on the corresponding 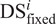 test sets rather than on variable intensity test sets, and (b) models trained on 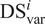 training sets show better performance on the corresponding test sets rather than on fixed intensity test sets. It also shows that 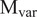 models are globally better than 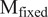 models. A plausible hypothesis is that training on variable intensities makes models better at generalizing on other data. Finally, 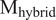 is the model that has the best overall performance, including the experimental dataset. Again, the diversity of training data drives this model to be more robust to newly encountered data.

More generally, given that the F1-score is above 90% for all the models, we can conclude that the architecture of the DeepSpot neural network is particularly suited for the task of mRNA spot enhancement.

### 4.4. DeepSpot enables more accurate spot detection compared with deepBlink

The main objective of our DeepSpot method is to circumvent the parameter fine-tuning and enable the downstream spot detection with a unique parameter set. As such, this objective fits well with the one that the authors of deepBlink^(^^7^ ^)^ have pursued, despite the fact that the latter proposes a new spot detection method, while our goal is to fit a spot enhancement step into commonly used workflows. Consequently, deepBlink constitutes a relevant comparison target.

**Figure 6.**
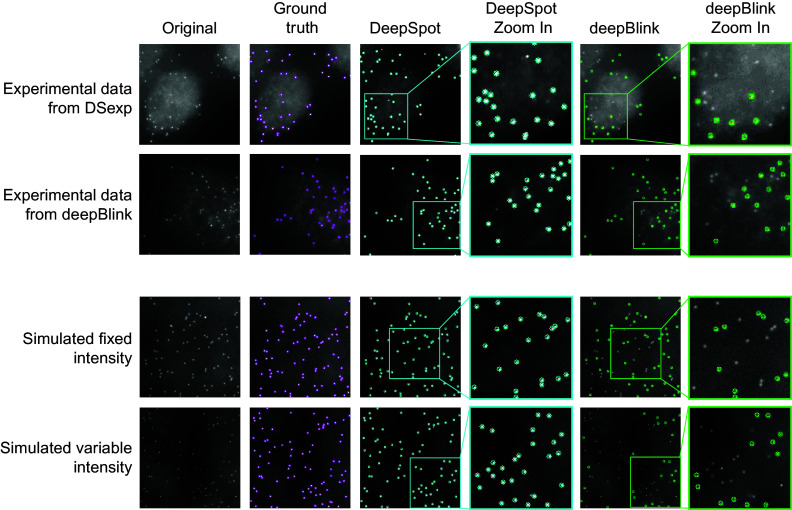
Examples of the results obtained with DeepSpot and deepBlink on the experimental test datasets DS_exp_ (first row) and deepBlink (second row), and the simulated spots with fixed (DS_fixed_) and variable (DS_var_) intensity datasets for the third and fourth rows, respectively. Colored circles indicate where the spots were detected by DeepSpot and deepBlink (blue and green, respectively). In the ground truth column, pink circles indicate the spots that were previously annotated as ground truth by alternative methods. The last two columns show the magnification at the positions indicated by the colored rectangles for DeepSpot and deepBlink, respectively.

We compared the accuracy of spot detection by the model 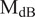 made available on deepBlink associated GitHub with that of DeepSpot when trained on hybrid data 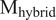 , both on our datasets as well as on the dataset provided by the authors of deepBlink, 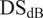 . Table 4 shows the F1-scores for each dataset category. It should be noted that deepBlink’s precision is close to 99%, while its recall is very low for certain datasets (Supplementary Figure S4), meaning that the main drawback of deepBlink is in terms of false negatives (missing true spots). We illustrate this in Figure 6, where we provide a comparison of deepBlink and DeepSpot on both experimental and simulated data examples.

**Table 4.** Models’ performance for deepBlink and DeepSpot for smFISH spot detection. Overall F1-scores are calculated by averaging the values obtained for each image of the test datasets corresponding to each dataset category. Top values in each cell correspond to the mean value, whereas bottom values between brackets show the 95% confidence interval. Best values are highlighted in bold.

F1-score (%) Model	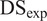	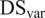	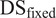	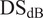
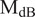	78.7	47.97	67.4	**94.2**
	[75.26, 82.14]	[46.86, 49.07]	[66.28, 68.51]	± 0.141 (std)
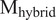	**94.43**	**96.67**	**98.04**	87.92
	[93.41, 95.45]	[96.58, 96.75]	[97.96, 98.12]	[85.36, 90.49]

DeepSpot clearly outperformed deepBlink on our datasets (Table 4). However, we found that 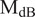 performed better on experimental data than on simulated data, presumably because deepBlink model has only been trained on experimental data. 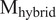 results were consistent on all of our datasets. We have also applied our 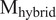 model to the smFISH test dataset 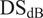 provided by the authors of deepBlink. Results reported in Table 4 for the detection of spots by 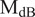 from the images of the 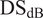 dataset are those obtained by the authors and originally reported in Reference (7). Not surprisingly, deepBlink model performance is better on their own smFISH images; however, DeepSpot managed to have an F1-score of nearly 88%, a noticeable achievement since the model has not been trained on the 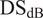 data. Together, the results of Table 4 indicated that DeepSpot is a robust methodology that offers a generalist model for mRNA spot enhancement and ensures high-quality downstream spot detection without parameter tuning.

### 4.5. DeepSpot’s use in an end-to-end smFISH experiment

**Figure 7.**
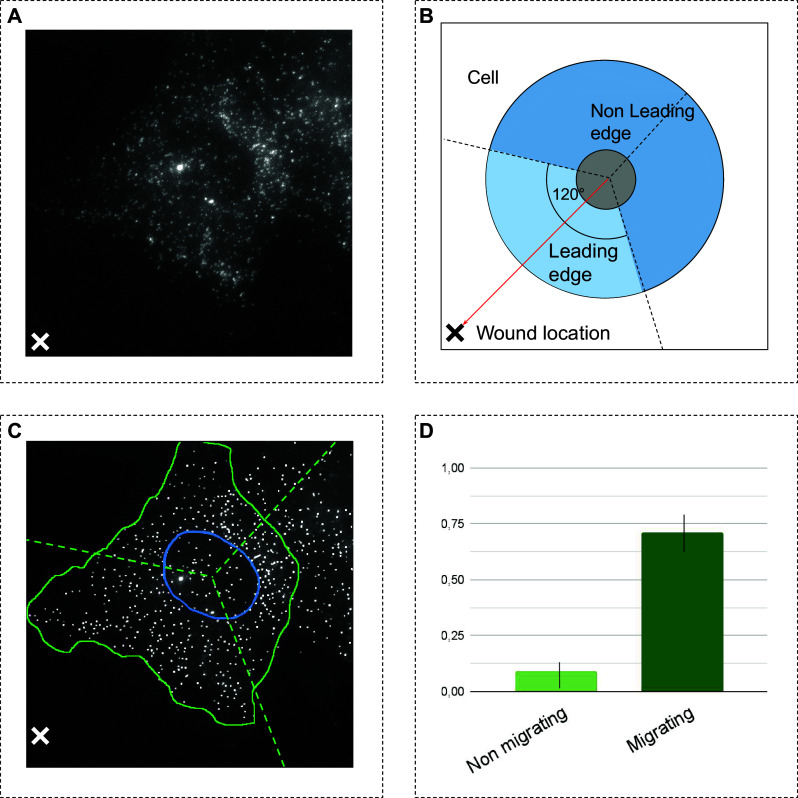
Processing steps for the end-to-end wound healing assay. Panel A shows a typical smFISH image of the wound healing assay. Panel B shows the cell quantization procedure in three sections and the direction of the wound (red arrow). 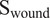 section is oriented toward the wound and is shown in light blue, and other sections are in dark blue. Panel C represents how the cell and nucleus were manually segmented and mRNA spots were counted after enhancement within the cytoplasmic portion of each section. Panel D presents the Wound Polarity Index (WPI) of cytoplasmic mRNA transcripts in 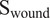 compared to other sections for the 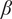 *-Actin* RNA. WPI was calculated in migrating and nonmigrating cells. The bars correspond to the median and the error bars to the standard deviation from the median for 100 bootstrapped WPI estimates.

To evaluate whether DeepSpot can be effectively used in an end-to-end smFISH experiment, we have performed a wound healing assay in which cells migrate toward a wound to close it. To investigate whether 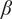 *-Actin* was enriched at the leading edges of 3T3 migrating fibroblast cells (see Section 3.1.2), the wound location was manually annotated as shown in a typical image example in Panel A of Figure 7.

All the cell images were segmented by manually, yielding cell and nucleus masks. Wound location defined the cell migration direction as schematically shown in Panel C of Figure 7. We partitioned the cell masks into three sections by computing 120° section centered at the nucleus centroid, and anchoring one of these sections as oriented toward the wound location 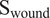 at 60° angle to the left and to the right of the line between the nucleus centroid and the wound location. This cell segmentation allowed us to compute the normalized number of detected mRNA spots in the cytoplasmic part of each section 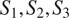 .

Using the DypFISH framework^(^^44^ ^)^, we further compared the cytoplasmic mRNA relative density in 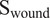 (light blue) and in sections that are not oriented toward the wound (dark blue), as shown in Panel B of Figure 6. We used the Polarity Index^(^^44^ ^)^, that measures the enrichment of mRNA in different sections. Briefly, the Polarity Index measures how frequently the relative concentration within the wound section is higher than in the non-wound section. The Polarity Index lies between 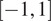 : a positive value implies a wound-correlated enrichment of RNA transcripts, whereas a negative value implies enrichment away from the wound, and a value of zero implies no detectable enrichment.


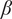
 *-Actin* mRNA was highly enriched in the leading edge of migrating cells, whereas almost no detectable enrichment of 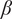 *-Actin* was found in the leading edge of control cells. This is in line with previously published data, showing enrichment of 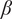 *-Actin* in leading edges of migrating fibroblasts^(^^45^ ^)^.

## 5. Discussion and Conclusion

Recent FISH microscopy methods are capable of generating thousands of images, and it has thus become imperative to introduce algorithms capable to streamline the detection of mRNA spots and in particular to avoid manual fine-tuning of numerous parameters.

### 5.1. Limitation

The impact of the nonspecifically bound probes was not evaluated in this work. Consequently, even if DeepSpot is trained to distinguish between multiple signal intensity variations, in cases where the signal intensity of the nonspecifically bound probes is too similar in shape and intensity to the signal of the specifically bound probes, then DeepSpot will not be able to enhance only spots corresponding to specifically bound probes. This issue, common to all detection methods, deserves further investigation.

Moreover, it remains to be investigated whether DeepSpot would be suitable for more complex systems such as tissues and which adaptations in terms of its architecture and training data such improvement would require. Moreover, even if DeepSpot architecture was designed to be easily extensible to 3D by changing the 2D convolution layers to 3D layers in TensorFlow, the performance of DeepSpot in 3D has not been evaluated.

### 5.2. Discussion

In this work, we introduced DeepSpot, a novel CNN architecture specifically designed to enhance RNA spots in FISH images, thus enabling the downstream use of well-known spot detection algorithms, such as the Icy spot detector, without parameter tuning. In particular, the architecture of our network introduces the CASO module that relies on sparse convolution to provide more context for enhancement of small objects corresponding to mRNA spots. DeepSpot network has been trained and tested on 21 simulated datasets, all with different signal and noise characteristics, as well as on a previously published experimental dataset that was annotated for spot locations. We have shown that (a) our approach achieves better performance when the training is performed on data with highly variable intensity and (b) performing training on a combination of experimental and simulated data is a viable approach in real-life setting.

Furthermore, we compared the performance of combining DeepSpot and Icy to that of the state-of-the-art deep-learning-based method deepBlink and have shown that, on average, DeepSpot enables a substantially better detection of mRNA spots than deepBlink. We found that DeepSpot/Icy workflow provided excellent quality spot detection on the test datasets corresponding to the datasets on which it has been trained, with the average F1-score of above 97%, but also achieved high-precision results on fully unknown datasets with the F1-score of 88% for the datasets provided with the deepBlink publication. Taken together, the good results on both known and unknown data indicate that DeepSpot is a more generalist model than deepBlink and that it achieves a good balance between overfitting and underfitting. We hypothesize that this generalization capacity is possibly due to both strong regularization within the network and the diversity of signal provided by the carefully constructed training data.

To evaluate how well our method is suited for end-to-end biological investigations, we have shown the efficiency of the DeepSpot model trained on the combination of experimental and simulated data in the context of an independent study of cell migration. We have performed smFISH to detect 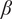 *-Actin* in mouse fibroblasts in a wound healing assay and enhanced the resulting images using our combination model, which allowed us to detect that the 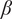 *-Actin* mRNA enrichment is specific to leading edge of migrating cells as contrasted by its expression in nonmigrating cells.

To conclude, we have shown that DeepSpot enhancement enables automated detection and accurate localization of mRNA spots for downstream analysis methods and can thus be a useful tool to streamline not only spot detection, but also studies of localized mRNA enrichment within cells.

## Data Availability

DeepSpot network along with the code for training and for mRNA spot enhancement is fully open source and available on GitHub at https://github.com/cbib/DeepSpot. Our pretrained models used in this study are also available on our GitHub page, as well as the Napari plug-in. Data for simulated images for 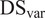 , 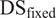 , and 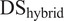 are available on Xenodo at https://doi.org/10.5281/zenodo.5724466.

## Hardware and Framework

All the analysis presented in this paper are performed on 1 GPU Tesla T4 with 16 GB of memory, on a dedicated machine with two 2 CPU Intel Xeon Silver 4114 and 128 GB RAM. Training curves and comparison with a vanilla U-Net architecture are available in Supplementary Figure S5 and Supplementary Table S1. This work is implemented in Python 3.8, and we used TensorFlow 2.4 for creating and training the neural networks.

## Supplementary material

For supplementary material accompanying this paper visit https://doi.org/10.1017/S2633903X22000034. click here to view supplementary material
