# Supplementary material for: DeepSpot: A deep neural network for RNA spot enhancement in single-molecule fluorescence in-situ hybridization microscopy images
Source: Biol Imaging. 2022 Apr 19;2:e4. doi: 10.1017/S2633903X22000034 (PMC10951802; doi:10.1017/S2633903X22000034)
Supplement: Supplementary file 1 [file S2633903X22000034sup001.pdf]

Supplementary Information (SI)

DeepSpot: a deep neural network for RNA spot enhancement in smFISH microscopy images

Authors : Emmanuel Bouilhol*, Anca Flavia Savulescu, Edgar Lefevre, Benjamin Dartigues, Robyn Brackin and Macha Nikolski*

*To whom correspondence should be addressed: emmanuel.bouilhol@gmail.com; macha.nikolski@u-bordeaux.fr

Supplementary Figures S1-5
Supplementary Table S1

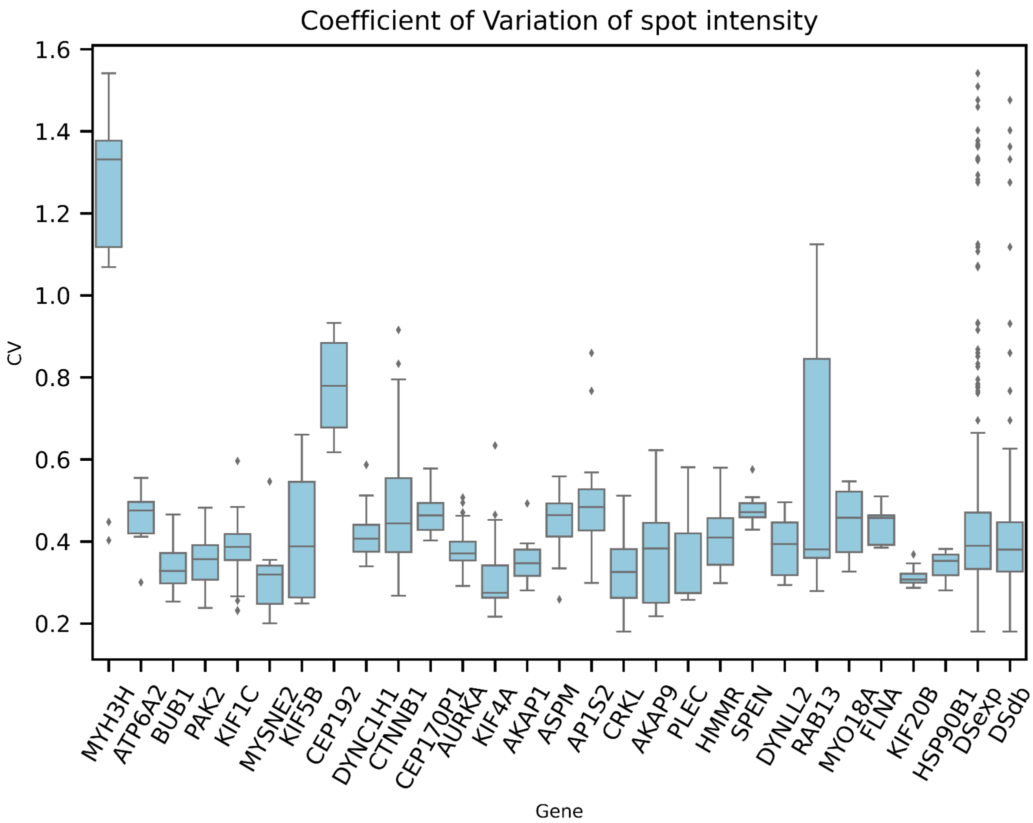

Supplementary Figure S1: Boxplots of the Coefficients of Variation of the spots intensities (defined as the ratio $\frac{\sigma}{\mu}$) for 27 genes and for the whole dataset DSexp (Chouaib et al., 2020). Maximum intensity of each spot from DSexp annotated by Chouaib et al. (2020) and DSdb annotated by Eichenberger et al., (2021) is used to compute the Coefficient of Variation (CV) of spot intensities.

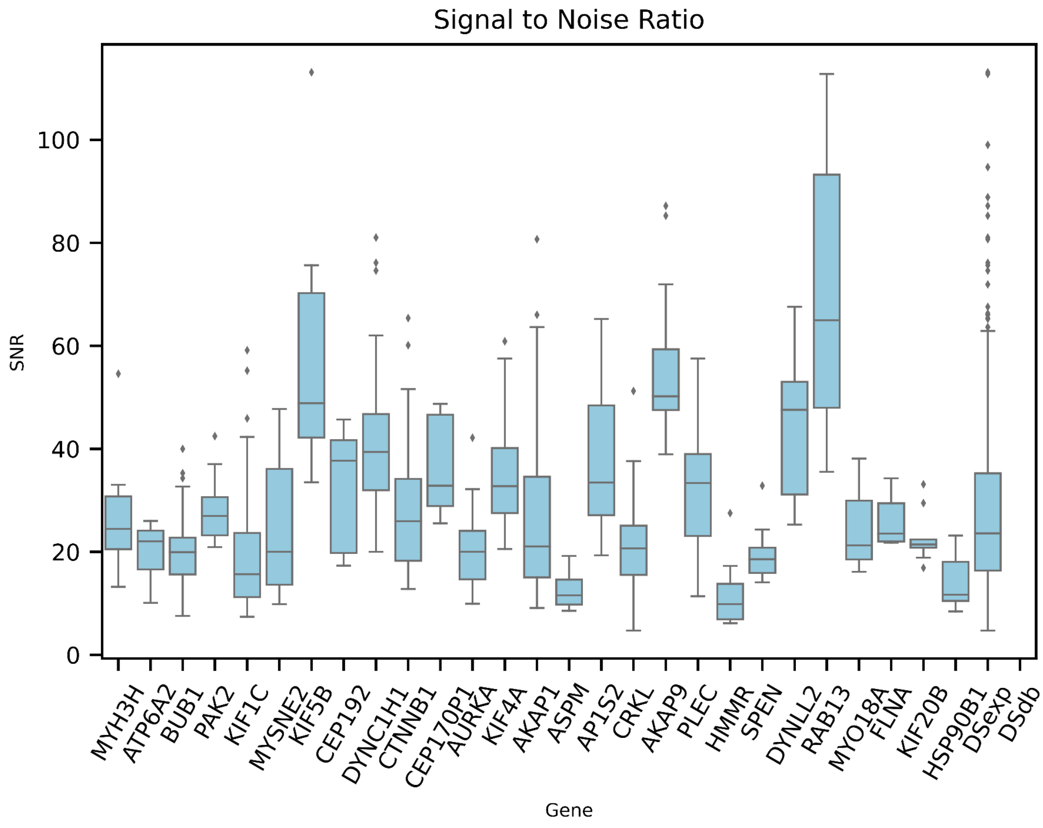

Supplementary Figure S2: Signal to Noise Ratio (SNR) distributions. SNR is measured by $SNR = (S - B) / \sigma$, where S is the maximum pixel intensity of spots in an image, B is the mean value of the background and σ is the standard deviation of the background. SNR is computed for each image; boxplots represent the distribution of SNR for each of the 27 genes, for the entire curated dataset (DSexp) from the Chouaib et al., (2020) publication and the dataset (DSdb) from Eichenberger et al., (2021).

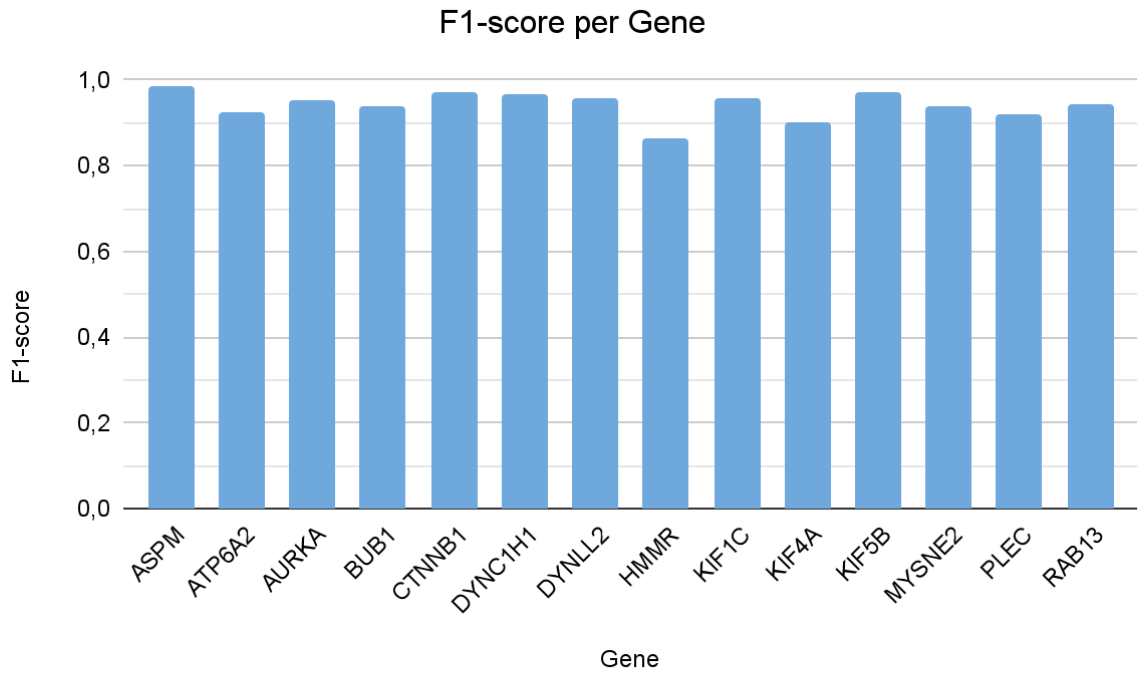

Supplemental Figure S3: F1-score of spot detection after DeepSpot enhancement for 14 genes belonging to the test dataset from Chouaib et al. (2020). For each gene, F1-scores were calculated by averaging the F1-score obtained for each image.

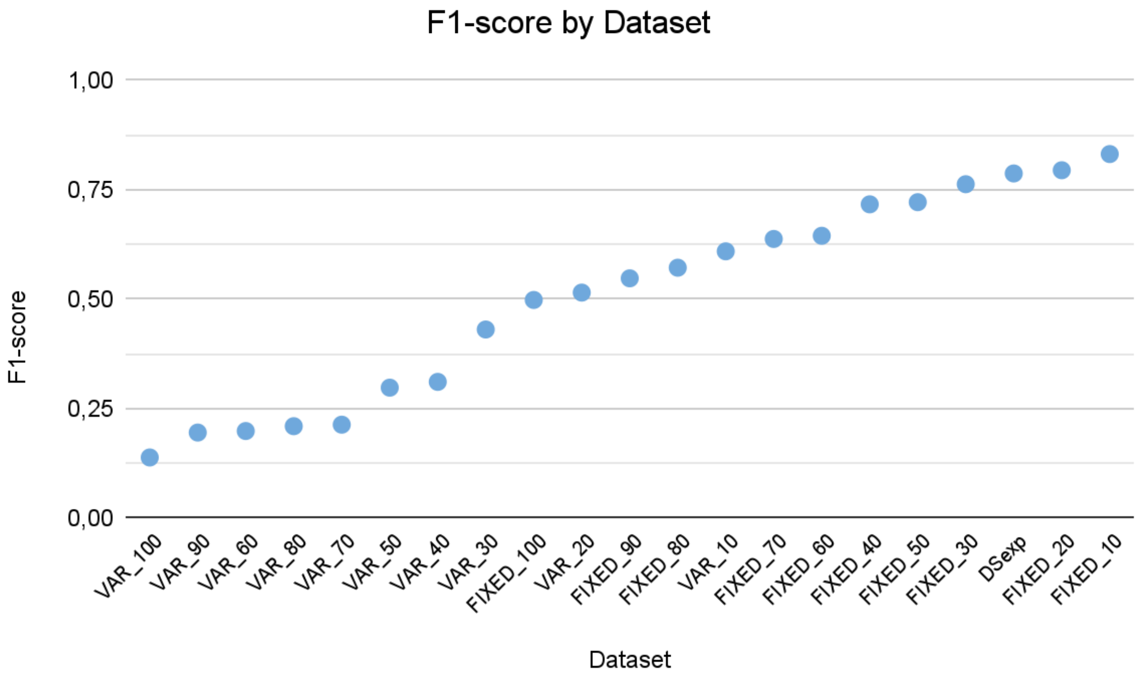

Supplemental Figure S4: F1-Scores of deepBlink spot detection on all simulated and experimental datasets. Scores were obtained by averaging the F1-score values of all images belonging to each dataset.

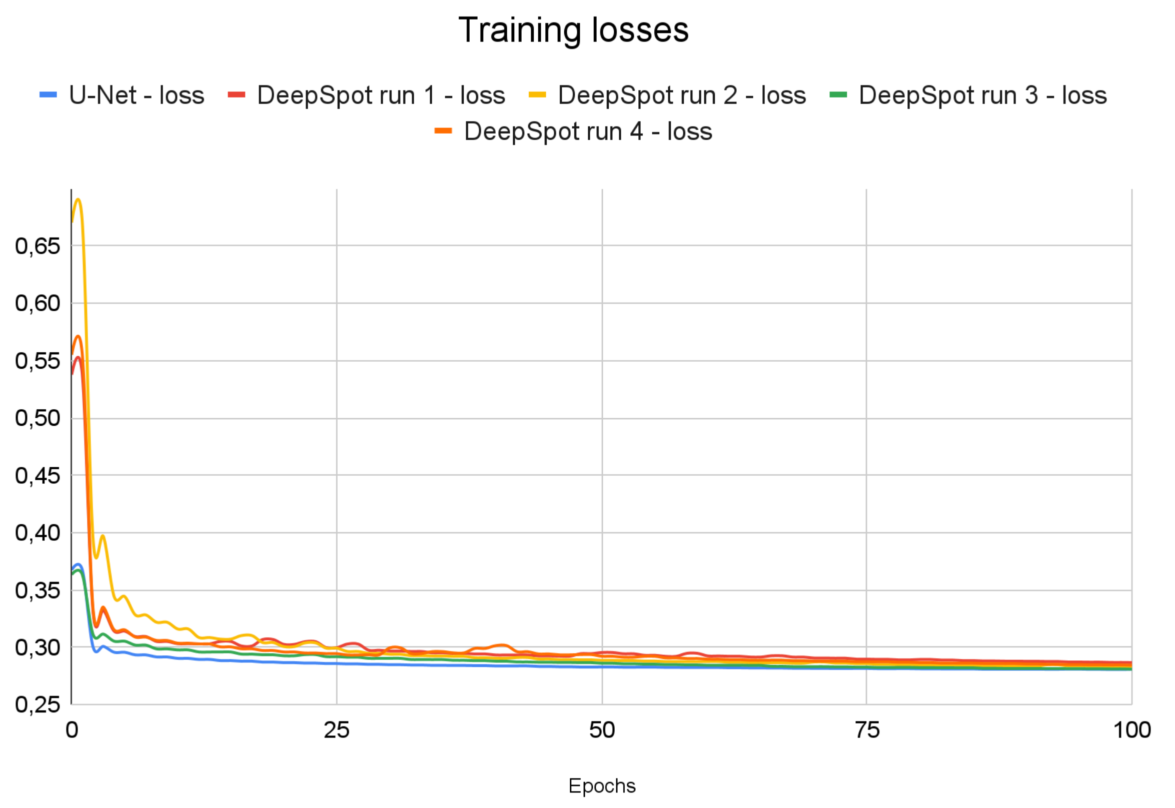

Supplemental Figure S5: Loss values per epoch for 4 different trainings of DeepSpot and one training of a vanilla U-Net. The training curve of the U-Net is shown in blue. Differences in the number of epochs are due to the early-stopping. All trainings were performed with the same parameters, namely a patience of 50 epochs on the Nvidia T4 GPU cards.

Training	Epochs	Execution time (minutes)
U-Net	342	232
DeepSpot run 1	268	397
DeepSpot run 2	264	380
DeepSpot run 3	213	316
DeepSpot run 4	265	386

Supplemental Table S1: Benchmarking execution time for DeepSpot and a vanilla U-Net. All five trainings were performed with the same setup described in the manuscript in the "Hardware and Framework" section. Differences in the number of epochs are due to the use of early-stopping. All trainings were run with the same parameters, namely a patience of 50 epochs on the Nvidia T4 GPU cards.
